# Changes in Muscle and Cerebral Deoxygenation and Perfusion during Repeated Sprints in Hypoxia to Exhaustion

**DOI:** 10.3389/fphys.2017.00846

**Published:** 2017-10-31

**Authors:** Sarah J. Willis, Laurent Alvarez, Grégoire P. Millet, Fabio Borrani

**Affiliations:** Faculty of Biology and Medicine, Institute of Sport Sciences, University of Lausanne, Lausanne, Switzerland

**Keywords:** repeated sprint ability, altitude, oxygenation, NIRS, maximal exercise, convection, diffusion, blood flow

## Abstract

During supramaximal exercise, exacerbated at exhaustion and in hypoxia, the circulatory system is challenged to facilitate oxygen delivery to working tissues through cerebral autoregulation which influences fatigue development and muscle performance. The aim of the study was to evaluate the effects of different levels of normobaric hypoxia on the changes in peripheral and cerebral oxygenation and performance during repeated sprints to exhaustion. Eleven recreationally active participants (six men and five women; 26.7 ± 4.2 years, 68.0 ± 14.0 kg, 172 ± 12 cm, 14.1 ± 4.7% body fat) completed three randomized testing visits in conditions of simulated altitude near sea-level (~380 m, F_I_O_2_ 20.9%), ~2000 m (F_I_O_2_ 16.5 ± 0.4%), and ~3800 m (F_I_O_2_ 13.3 ± 0.4%). Each session began with a 12-min warm-up followed by two 10-s sprints and the repeated cycling sprint (10-s sprint: 20-s recovery) test to exhaustion. Measurements included power output, vastus lateralis, and prefrontal deoxygenation [near-infrared spectroscopy, delta (Δ) corresponds to the difference between maximal and minimal values], oxygen uptake, femoral artery blood flow (Doppler ultrasound), hemodynamic variables (transthoracic impedance), blood lactate concentration, and rating of perceived exertion. Performance (total work, kJ; −27.1 ± 25.8% at 2000 m, *p* < 0.01 and −49.4 ± 19.3% at 3800 m, *p* < 0.001) and pulse oxygen saturation (−7.5 ± 6.0%, *p* < 0.05 and −18.4 ± 5.3%, *p* < 0.001, respectively) decreased with hypoxia, when compared to 400 m. Muscle Δ hemoglobin difference ([Hbdiff]) and Δ tissue saturation index (TSI) were lower (*p* < 0.01) at 3800 m than at 2000 and 400 m, and lower Δ deoxyhemoglobin resulted at 3800 m compared with 2000 m. There were reduced changes in peripheral [Δ[Hbdiff], ΔTSI, Δ total hemoglobin ([tHb])] and greater changes in cerebral (Δ[Hbdiff], Δ[tHb]) oxygenation throughout the test to exhaustion (*p* < 0.05). Changes in cerebral deoxygenation were greater at 3800 m than at 2000 and 400 m (*p* < 0.01). This study confirms that performance in hypoxia is limited by continually decreasing oxygen saturation, even though exercise can be sustained despite maximal peripheral deoxygenation. There may be a cerebral autoregulation of increased perfusion accounting for the decreased arterial oxygen content and allowing for task continuation, as shown by the continued cerebral deoxygenation.

## Introduction

During exercise, the circulatory system is challenged to improve oxygen delivery of the working tissues. Oxygen delivery is known to be regulated by physiological mechanisms of both convective (as decrease in arterial oxygen content in hypoxic conditions due to a lower inspired fraction of oxygen; di Prampero, 2003; Sweeting et al., 2017; Villar and Hughson, 2017) and diffusive (as blood flow is regulated via vascular vasodilation based on the metabolic demand of exercise and transports to the muscle for perfusion and to the mitochondria for energy metabolism; Calbet, 2000; di Prampero, 2003; Laughlin and Joyner, 2003; Walker et al., 2007) factors. It has been demonstrated that diminishing the oxygen availability has a detrimental effect on endurance performance as well as on the ability to repeat maximal and short sprints without adequate recovery (Balsom et al., 1994; Smith and Billaut, 2010, 2012; Girard et al., 2011; Billaut and Buchheit, 2013).

As exercise intensity increases, there is a progressive muscle deoxygenation to a minimal point or plateau close to maximal power output or exhaustion (Grassi et al., 1999; Neary et al., 2001; Subudhi et al., 2007). In hypoxic conditions, this plateau in deoxyhemoglobin concentration has been considered an indication of maximal skeletal muscle oxygen extraction as a product of reduced oxygen availability (Esaki et al., 2005; Legrand et al., 2005). The peripheral muscle tissue was shown to regulate this oxygen extraction in order to longer maintain the balance between oxygen delivery and consumption in hypoxic conditions (Subudhi et al., 2007; Smith and Billaut, 2010, 2012). Researchers have also reported that maintaining a high muscle oxygen extraction during low arterial oxygen pressure conditions facilitates the development of hypoxemia in trained individuals (Van Thienen and Hespel, 2016).

Systemic hypoxemia is considered to be a strong contributor to cerebral deoxygenation, (Nielsen et al., 2002; Amann and Calbet, 2008; Amann and Kayser, 2009) which has been shown to occur also during repeated sprint exercise (Billaut and Smith, 2010). Cerebral deoxygenation has been considered as an influential factor for decreasing exercise intensity or cessation of exercise (Smith and Billaut, 2010). Furthermore, low brain oxygenation due to insufficient oxygen delivery and/or lower pressure gradient of arterial oxygen has an influence on the diffusive delivery of oxygen to the sarcomere and mitochondria, which may induce a central fatigue (Amann and Calbet, 2008). The arterial blood pressure (i.e., cerebral perfusion pressure) increases during exercise, and with rapid or forceful muscle contractions may exceed the limits of cerebral autoregulation (maintenance of blood flow to the brain under conditions of changing blood pressure), thus exposing a potential risk of high blood pressure combined with increased blood flow in the brain (Bill and Linder, 1976; MacDougall et al., 1985; Calbet et al., 2015a; Curtelin et al., 2017). In addition, cerebral blood flow provides an important signal to the central nervous system (CNS) which may become a supplemental limiting factor for exercise at altitude in addition to cardiorespiratory capacity and muscle fatigue (Kayser, 2003; Imray et al., 2005). Moreover, the CNS is sensitive to the factors of partial pressure of arterial oxygen, arterial oxygen content, and arterial oxygen saturation, which together (along with many other factors) influence the cardiac output and thus have an impact on brain function (Calbet et al., 2003). Indeed, cerebral function during exercise (especially supramaximal intensity) may have a large influence on fatigue in addition to the decrease in peripheral muscle performance (Shibuya et al., 2004; Amann et al., 2007; Subudhi et al., 2007; Amann and Calbet, 2008; Smith and Billaut, 2010). However, there is still uncertainty regarding cerebral oxygenation and the effect on performance as well as cessation of exercise during repeated sprints in hypoxia.

Changes in oxygenation and the hemodynamics of these tissues can be non-invasively recorded using near-infrared spectroscopy (NIRS) for real-time measures (Van Beekvelt et al., 2001). Previous reports have examined peripheral and cerebral oxygenation with NIRS during repeated sprint exercise, however, none of these studies have been performed with repeated sprint exercise to exhaustion in hypoxic conditions [series of repeated sprints, (Racinais et al., 2007; Smith and Billaut, 2010, 2012; Billaut and Buchheit, 2013); or incremental ramp test to exhaustion, (Amann et al., 2007; Subudhi et al., 2007)]. In addition, recent research has eluded that there may be greater blood volume shifts in the muscle during repeated sprint exercise possibly due to greater arteriolar dilation together with increased capillary volume (De Smet et al., 2017). Therefore, there are factors contributing to the end of exercise within maximal repeated sprints to exhaustion which remain unknown.

Thus, the aim of the present study was to investigate changes in peripheral and cerebral oxygenation during maximal repeated sprints to exhaustion as well as the physiological effects of different levels of hypoxia. The hypothesis was that performance would be impaired in hypoxia due to decreased convective oxygen delivery. Additionally, the changes in peripheral and cerebral de/re-oxygenation during sprints/recoveries were hypothesized to be greater as hypoxia increased, and that cerebral oxygenation variables may challenge blood flow regulation at the end of exercise.

## Methods

### Participants

Eleven healthy, recreationally active volunteers participated in this study (six men and five women; 26.7 ± 4.2 years, 68.0 ± 14.0 kg, 172 ± 12 cm, 14.1 ± 4.7% body fat). Participants were required to train at least 4 h per week and be accustomed to maximal intensity exercise. Exclusion criteria for participation included any skeletal or muscular injury in the last 3 months, pain, or any other medical condition which could compromise the study. Participants gave written informed consent after being informed of the procedures and risks involved. The experimental protocol was approved by the Ethical Commission for Human Research CER-VD 138/15 and conducted according to the Declaration of Helsinki.

### Study design

In a randomized, single-blinded experimental protocol, participants reported to the laboratory for a total of four sessions (one familiarization and three testing visits). The testing visits were performed in simulated altitude of sea level (~380 m, F_I_O_2_ 20.9%), ~2000 m (F_I_O_2_ 16.5 ± 0.4%), and ~3800 m (F_I_O_2_ 13.3 ± 0.4%). Each session was completed in a normobaric hypoxic chamber (ATS Altitude Training, Sydney, Australia) built in the laboratory. This chamber utilizes a system containing a compressor connected to air filters which enable the regulation of the level of oxygen (simulated altitude settings as noted above). Participants were asked to avoid strenuous activity as well as caffeine or alcohol consumption 24 h before each visit. All visits were scheduled at the same time of day and at least 48 h apart to limit fatigue.

### Familiarization

During the familiarization visit, anthropometric data (body height, body mass, and skin fold measurement) were collected along with the completion of the informed consent and health questionnaires. Skin fold measurements were obtained by an experienced technician using the seven-site formula from the 2014 ACSM guidelines (ACSM, 2014). Afterwards, participants were seated on an electronically braked cycling ergometer (Lode Excalibur Sport Ergometer, Lode B.V., Netherlands) and dimensions were recorded for standardization during subsequent sessions. After a 5-min warm-up at 1.5 W·kg^−1^, participants performed two 10-s maximal sprints with 3 min of active recovery between. Following an additional 5-min passive recovery, participants were familiarized with the repeated sprint ability test (RSAT), as described in detail below.

### Testing visits

**Figure 1:**
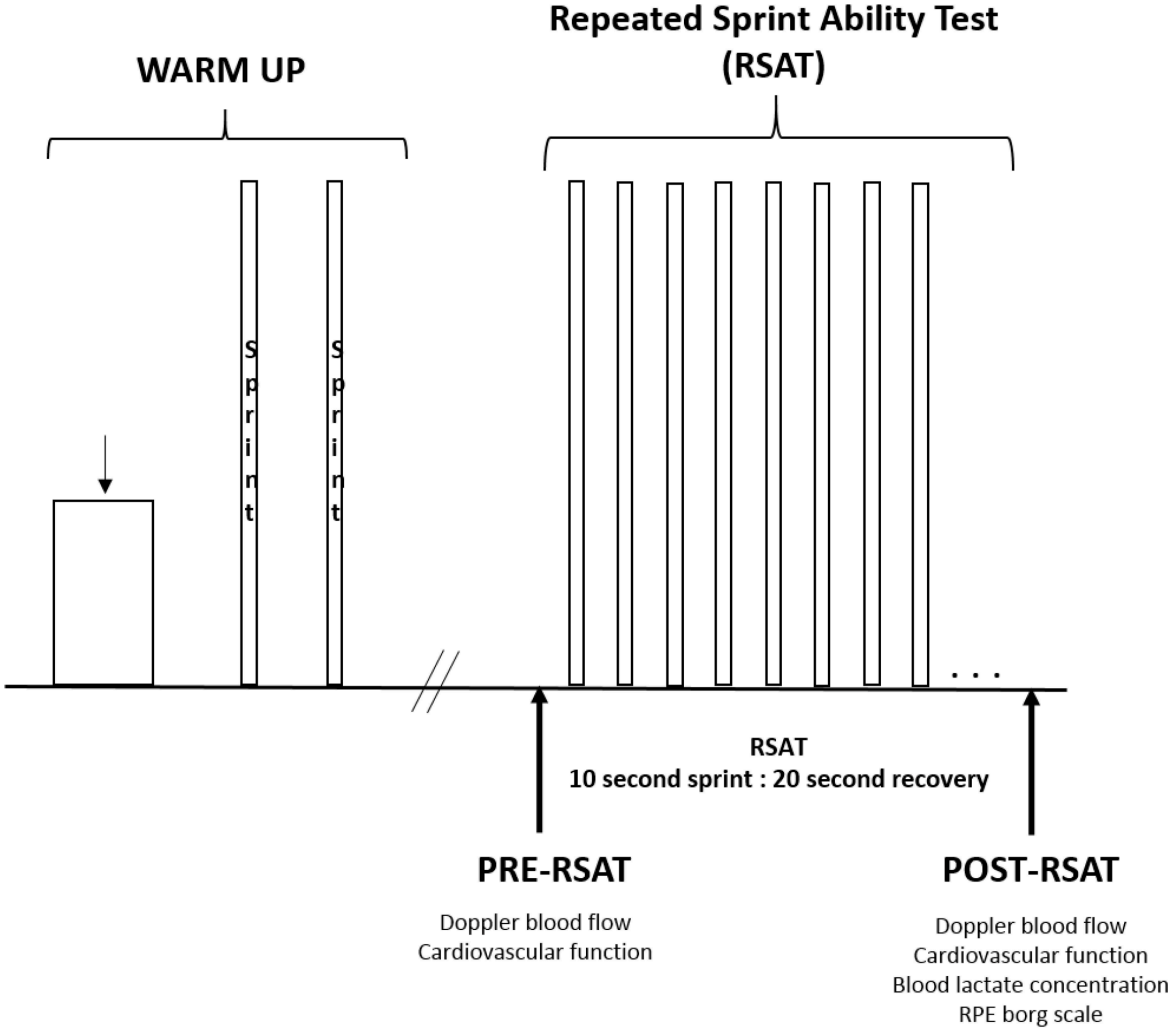
General protocol of warm up and repeated sprint ability test to exhaustion.

The protocol of the testing visit is illustrated in Figure 1. Each session began in the normobaric hypoxic chamber with a 12-min warm-up (6 min at 50 W, 6 min at 100 W) at a cadence of 85 rpm. Following which, the two maximal 10-s warm-up sprints were performed (similar to the familiarization visit) with 3 min of active recovery between sprints. After a 5-min passive recovery, the measurements for pre-RSAT were collected. These measurements (described in detail below) included resting Doppler blood flow and estimates of cardiovascular function synchronized at the same moment. Participants were then fitted with a mask to measure oxygen uptake. Participants then performed the RSAT to exhaustion. Upon completion of the test, measurements were taken in the following sequence: Doppler blood flow at 1-min from end of RSAT in synchronization with cardiovascular function, blood lactate concentration, and rating of perceived exertion (RPE) values.

### Repeated sprint ability test

After the normalization and warm-up period described above, participants performed the RSAT as previously described previously (Faiss et al., 2013). Participants began pedaling at 20 W with a cadence of 85 rpm for 1 min, followed immediately by the RSAT of 10-s all-out maximal sprint and 20-s active recovery (1:2 work-to-rest ratio) until volitional exhaustion or task failure (cadence <70 rpm) similar to Faiss et al. (2013). Immediately at the end of each 10-s sprint, the ergometer automatically switched to a resistance of 20 W for the 20-s recovery. Participants were given very strong verbal encouragement and were not given any indication of the number of sprints performed. All sprints were performed using the “Wingate mode” from the manufacturer with an individually fixed torque factor of 0.8 Nm·kg^−1^. Participants were instructed to perform each sprint as hard and fast as possible and perform as many sprints as possible while also maintaining a similar body position. In order to avoid any pacing strategy, the first two sprints were controlled to obtain at least 95% of the best sprint from the two warm-up sprints performed, which was the case for each test. Mean power (W), number of sprints performed, and total work (kJ) were obtained for further analysis, as well as the calculation of fatigue index or percent decrement (S_dec_(%) = [1 − ((S_1_ + S_2_ + S_3_ + … + S_final_)/(S_best_ × number of sprints))] × 100), where S_1_ corresponds to sprint 1, etc., (Glaister et al., 2008). During RSAT, pulse oxygen saturation was measured at the earlobe with an oximeter (8000Q2 Sensor, Nonin Medical Inc., Amsterdam, The Netherlands) and recorded at 5 Hz, the minimum of which was used for further analysis. The RPE was evaluated using the Borg scale (6–20) as a perception of effort in both the legs and the breathing immediately after the test.

### Metabolic measurements

Breath-by-breath pulmonary gas-exchange data were collected continuously (Medgraphics CPX, Loma Linda, CA, USA). Oxygen consumption ($\overset{\cdot }{\text{V}}$O_2_), ventilation ($\overset{\cdot }{\text{V}}$_E_), ventilatory equivalent for oxygen ($\overset{\cdot }{\text{V}}$_E_/$\overset{\cdot }{\text{V}}$O_2_), and for carbon dioxide ($\overset{\cdot }{\text{V}}$_E_/$\overset{\cdot }{\text{V}}$CO_2_), respiratory exchange ratio (RER), and breath frequency (BF) were computed and stored for further analyses. The system was calibrated with a 3-L syringe (M9474, Medikro Oy, Finland) and known gas mixtures of O_2_ and CO_2_ prior to each measurement. Heart rate was monitored at 5 Hz with a telemetry based heart rate monitor (Polar RS400, Kempele, Finland) and the maximum recorded was used for further analysis. During the RSAT, the highest 30-s average of oxygen uptake was computed and used for further analysis. In addition, blood lactate concentration was assessed at the earlobe post-RSAT. After the skin was cleaned and dried, a lancet was used to take a small droplet (0.2 μl) into the strip for analysis (Lactate Scout, EKF Diagnostics, GmbH, Leipzig, Germany).

### Cardiovascular measurements

Estimates of cardiovascular variables were obtained throughout the entire protocol using Physioflow® (Manatec type PF05L1, Paris, France). The measuring device is based on a bioimpedance method which continuously calculates stroke volume (SV), heart rate (HR), cardiac output (Q), systemic vascular resistance (SVR), end diastolic volume (EDV), and ejection fraction (EF) to detect changes in transthoracic impedance during cardiac ejection. These parameters were analyzed at the same moment (average of 30 s) as the pre- and post- Doppler blood flow measurements. While in the cycling position at rest, Doppler blood flow measurements were collected by an experienced technician on the left femoral artery with a linear probe (L12-5L60N) using EchoWave II 3.4.4 software (Telemed Medical Systems, Telemed Ltd. Lithuania, Milano, Italy) ~5 min pre-, as well as at 1-min post-RSAT. A video image was obtained for 30 s and subsequent analysis was performed to take an average of 10 frames, meaning a measurement every ~1.5 s. Measurements of the blood flow were calculated within the software via a measurement of the vessel diameter (mm) and the blood velocity (cm·s^−1^).

### Near-infrared spectroscopy measurements

Muscle oxygenation was evaluated using the near-infrared spectroscopy (NIRS) technique as described previously by Boushel and Piantadosi (2000). The PortaMon and PortaLite devices (Artinis, Zetten, The Netherlands) were used to measure muscle oxygenation of the vastus lateralis (PortaMon) and of the prefrontal cortex (PortaLite) at wavelengths between 760 and 850 nm. All devices were placed into a tight transparent plastic wrap to avoid humidity and create a waterproof barrier for proper function and signal quality. The PortaMon was placed on the lower third of the vastus lateralis and attached with double sided tape, then wrapped with tension against the leg to reduce movement during exercise. The position was marked with a permanent pen and images were taken to reproduce the placement in subsequent visits. The PortaLite was attached on the surface of the left prefrontal cortex with double sided tape, then the subject was fitted with a head wrap to create a dark environment and maintain a stable position of the probe. Measurements included a standard differential pathlength factor of 4.0 for the vastus lateralis as there is a lack of any clear standard value for the quadriceps during cycling sprints (Faiss et al., 2013) and 6.0 for the prefrontal cortex, similar to van der Zee et al. (1992) and Amann et al. (2007). All signals were recorded at the maximum frequency for each device (10 Hz for PortaMon and 50 Hz for PortaLite) and then exported at 10 Hz for further analysis (Oxysoft 3.0.53, Artinis, The Netherlands). For analysis, a 4th-order low-pass zero-phase Butterworth filter (cutoff frequency 0.2 Hz) was implemented to reduce artifacts and smooth perturbations in the signal from pedal strokes. Detection of maximum and minimum was performed automatically using deoxyhemoglobin as reference value for the start point of sprints. This allowed determination of successive sprint and recovery phases to be identified, and sprint phases to be further analyzed. The change (Δ) for each sprint was defined as the difference between maximum and minimum values for each sprint. Delta concentrations of oxyhemoglobin (Δ[O_2_Hb]), deoxyhemoglobin (Δ[HHb]), hemoglobin difference (Δ[Hbdiff]), total hemoglobin (Δ[tHb]), and tissue saturation index (ΔTSI, %) were obtained. Finally, the analysis was normalized to the duration of the set to exhaustion; i.e., percentage of sprints performed (i.e., 20, 40, 60, 80, 100%), and a linear interpolation was calculated when there was a fractional number of sprints, since each participant performed a different number of sprints in each condition.

### Statistical analysis

Cardiovascular estimates and blood flow measurements were evaluated with a linear mixed effects analysis of the relationship between condition (400, 2000, 3800 m) and time (pre or post). Fixed effects included condition and time, while participant was set as a random effect. Measurements of oxygenation were also evaluated with a linear mixed model analysis with fixed effects of condition and set duration (20, 40, 60, 80, 100% of sprints performed) with participants as the random effect. The remaining variables including: performance, metabolic gas exchange, pulse oxygen saturation, blood lactate, and RPE were also analyzed with a linear mixed model setting condition as the fixed effect and participant as the random effect. Visual inspection of residual plots did not reveal obvious deviations from homoscedasticity or normality. All analyses were performed using R (R Core Team, 2017, Foundation for Statistical Computing, Vienna, Austria) and nlme4 (Pinheiro et al., 2017). *P*-values were set to 0.05 and were obtained by likelihood ratio tests of the full model with the effect in question against the model without the effect in question. Contrasts were obtained using least-squares means for mixed models [library lsmeans, (Lenth, 2016)] employing Tukey method. Values are represented in tables and figures as mean ± standard deviation.

## Results

### Performance

All performance data are presented in Table 1. The number of sprints performed to exhaustion decreased by 26.9 ± 29.5% at 2000 m (*p* < 0.05) and 44.9 ± 22.8% at 3800 m (*p* < 0.001) when compared to 400 m. Similarly, the total work was decreased by 27.1 ± 25.8% (*p* < 0.01) and 49.4 ± 19.3% (*p* < 0.001), respectively. As expected, the pulse oxygen saturation also decreased 7.5 ± 6.0% at 2000 m (*p* < 0.05) and 18.4 ± 5.3% at 3800 m (*p* < 0.001), when compared to 400 m.

**Table 1:** Performance and respiratory values during repeated sprint test to exhaustion in simulated altitude of 400, 2000, and 3800 m.

	**400 m**	**2000 m**	**3800 m**
Number of sprints	29.8 ± 13.7	19.8 ± 10.2^#^	15.4 ± 9.5^###^
	34 (13–47)	38 (9–47)	26 (7–33)
Mean power (W)	543 ± 135	557 ± 150	511 ± 139^&^
	403 (332–736)	456 (344–801)	480 (320–800)
Fatigue index (% decrement)	26.5 ± 7.2	23.6 ± 7.1	26.4 ± 8.0
	22 (14–36)	21 (13–34)	28 (12–40)
Total work (kJ)	162 ± 81	107 ± 41^##^	78 ± 48^###^
	256 (50–306)	137 (31–168)	25 (166–191)
Maximal heart rate (bpm)	185 ± 9	183 ± 11	178 ± 8^###^^&^
	29 (174–203)	34 (165–199)	25 (166–191)
SpO_2_ (%)	93.8 ± 4.5	86.7 ± 6.4^#^	76.5 ± 6.0^###^^&&&^
	14 (86–100)	18 (78–96)	18 (70–88)
$\overset{\cdot }{\text{V}}$O_2_ (ml·kg^−1^·min^−1^)	40.1 ± 4.1	36.4 ± 4.3^###^	32.0 ± 3.7^###^^&&&^
	14.0 (34.5–8.5)	11.5 (30.3–41.8)	9.8 (27.0–36.8)
RER	1.11 ± 0.06	1.19 ± 0.08^##^	1.22 ± 0.09^###^
	0.19 (1.01–1.20)	0.22 (1.10–1.32)	0.26 (1.06–1.32)
$\overset{\cdot }{\text{V}}$_E_ (L·min^−1^)	137 ± 28	137 ± 30	135 ± 35^#^^&^
	98.8 (93.6–192.4)	108.2 (90.6–198.8)	117.2 (80.8–198)
$\overset{\cdot }{\text{V}}$_E_/$\overset{\cdot }{\text{V}}$O_2_	50.7 ± 4.5	56.0 ± 4.8^###^	60.0 ± 6.6^###^^&&^
	13.1 (43.1–56.2)	14.4 (48.3–62.7)	21.9 (48.4–70.3)
$\overset{\cdot }{\text{V}}$_E_/$\overset{\cdot }{\text{V}}$CO_2_	45.8 ± 4.4	47.0 ± 4.1	49.3 ± 3.2^#^
	12.4 (39.9–52.3)	11.9 (40.4–52.3)	8.8 (45.0–53.8)
BF (br·min^−1^)	66.9 ± 5.0	64.2 ± 3.7	63.9 ± 6.5
	16 (59–75)	13 (58–71)	22 (54–76)
RPE legs (Borg 6-20)	17.7 ± 2.1	18.5 ± 1.3	17.8 ± 1.9
	5 (15–20)	4 (16–20)	6 (14–20)
RPE breathing (Borg 6-20)	18.3 ± 1.4	19.2 ± 0.8	18.3 ± 2.3
	4 (16–20)	2 (18–20)	8 (12–20)
Blood lactate (mmol·L^−1^)	9.5 ± 5.2	11.6 ± 4.8	10.7 ± 5.2
	16.9 (4.6–21.5)	14.9 (6.2–21.1)	13.7 (4.6–18.3)

*Mean ± SD. Range (minimum-maximum)*.

### *p < 0.001*,

## *p < 0.01*,

# *p < 0.05 for difference with 400 m*.

&&& *p < 0.001*,

&& *p < 0.01*,

& *p < 0.05 for difference with 2000 m*.

*SpO_2_, pulse oxygen saturation; $\overset{\cdot }{V}$O_2_, oxygen uptake; RER, respiratory exchange ratio; $\overset{\cdot }{V}$_E_, minute ventilation; BF, breathing frequency; RPE, rating of perceived exertion*.

### Metabolic responses

Metabolic data are also presented in Table 1. During the repeated sprint to exhaustion, the peak oxygen uptake was reduced by 9.3 ± 5.5% at 2000 m (*p* < 0.001) and 19.8 ± 5.7% at 3800 m (*p* < 0.001), when compared to 400 m. There was a higher respiratory exchange ratio (RER, $\overset{\cdot }{\text{V}}$CO_2_/$\overset{\cdot }{\text{V}}$O_2_) with increased altitude (*p* < 0.01). In addition, the ventilatory equivalent for oxygen ($\overset{\cdot }{\text{V}}$_E_/$\overset{\cdot }{\text{V}}$O_2_) increased by 10.6 ± 4.6% at 2000 m and 17.9 ± 5.0% at 3800 m, (*p* < 0.001), respectively.

### Cardiovascular responses

There was a main effect of time (pre-post) for stroke volume (SV, *p* < 0.05), heart rate (HR, *p* < 0.001), and cardiac output (Q, *p* < 0.001) which all increased, while the systemic vascular resistance (SVR) was decreased (*p* < 0.001). There were no main effects of condition and no interactions present. All cardiovascular results are shown in Table 2. The average increase in femoral artery blood flow from pre- to post- repeated sprint to exhaustion was 71% at 400 m, 34% at 2000 m, and 24% at 3800 m when compared with the resting measurement pre-RSAT.

**Table 2:** Average cardiovascular values pre- and 1-min post- repeated sprint ability test (RSAT) representing a main effect difference post-RSAT in simulated altitude of 400, 2000, and 3800 m.

	**400 m**		**2000 m**		**3800 m**	
	**Pre-**	**Post-**	**Pre-**	**Post-**	**Pre-**	**Post-**
SV	89 ± 11	101 ± 22^*^	77 ± 16	92 ± 22^*^	94 ± 18	98 ± 21^*^
	29 (74–103)	63 (73–136)	52 (52–104)	61 (69–130)	62 (74–136)	63 (76–139)
HR (bpm)	94 ± 17	136 ± 16^***^	97 ± 16	130 ± 25^***^	95 ± 16	123 ± 15^***^
	49 (72–121)	62 (108–170)	55 (69–124)	71 (107–178)	58 (67–125)	44 (101–145)
Q (L·min^−1^)	8.4 ± 2.0	14.0 ± 4.2^***^	7.6 ± 2.7	12.4 ± 5.5^***^	8.8 ± 1.9	12.0 ± 2.4^***^
	6.8 (5.4–12.2)	14.4 (7.9–22.3)	8.1 (4.8–12.9)	14.8 (8.4–23.2)	6.4 (6.3–12.7)	6.6 (8.9–15.5)
SVR (dyn.s·cm^−5^)	817 ± 173	508 ± 140^***^	999 ± 302	621 ± 182^***^	843 ± 165	611 ± 101^***^
	466 (588–1,054)	403 (324–727)	928 (582–1,510)	541 (338–879)	577 (623–1,200)	260 (508–768)
EDV (ml)	114 ± 18	134 ± 31^*^	107 ± 11	122 ± 22^*^	124 ± 32	121 ± 25^*^
	51 (89–140)	90 (91–181)	30 (94–124)	62 (98–160)	99 (95–194)	77 (96–173)
EF (%)	78.7 ± 3.3	77.6 ± 14.2	71.6 ± 12.7	75.6 ± 2.9	76.9 ± 8.3	81.0 ± 3.7
	8.8 (73.6–82.4)	50.8 (40.8–91.6)	37.5 (46.4–83.9)	26.7 (58.9–85.6)	26.3 (64.7–91.0)	11.1 (74.1–85.2)
Blood Flow (ml·min^−1^)^£^	405 ± 276	694 ± 716	633 ± 555	847 ± 588	631 ± 699	782 ± 744
	771 (14–785)	1,990 (11–2,001)	1,836 (9–1,845)	1,626 (138–1,764)	2,013 (5–2,018)	1,913 (19–1,932)

*Mean ± SD Range (minimum-maximum). Measures were obtained at rest prior to RSAT (pre-), and at 1-min post-RSAT*.

£ *Blood flow for one subject was obtained from the popliteal artery, thus the range of values is high*.

*** *p < 0.001*,

* *p < 0.05 for difference with pre-*.

*SV, stroke volume; HR, heart rate; Q, cardiac output; SVR, systemic vascular resistance; EDV, end diastolic volume; EF, ejection fraction*.

### Peripheral oxygenation

**Figure 2:**
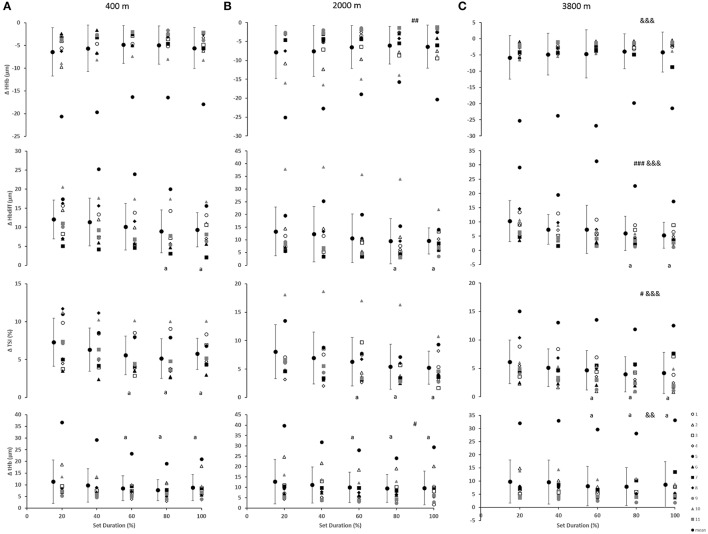
Near-infrared spectroscopy (NIRS) results representing the average maximum-minimum delta (Δ) value during the percentage of sprints completed to exhaustion for the individual response of the vastus lateralis in simulated altitude of **(A)** 400 m, **(B)** 2000 m, and **(C)** 3800 m. Mean ± SD. ^###^*p* < 0.001, ^##^*p* < 0.01, ^#^*p* < 0.05 for difference with 400 m; ^&&&^*p* < 0.001, ^&&^*p* < 0.01 for difference with 2000 m. Symbol: a for difference with 20%.

As indicated in Figure 2, for the vastus lateralis, there was a main effect of condition which resulted in a lower Δ[Hbdiff] and ΔTSI with the highest deoxygenation at 3800 m when compared with 400 and 2000 m (*p* < 0.05). Additionally, the main effect of condition was indicated for Δ[HHb] and Δ[tHb] in which resulted in greater changes in the 2000 m condition (*p* < 0.05) compared with 400 and 3800 m. There was a main effect of set duration near the end of the sprint test to exhaustion (*p* < 0.05) with Δ[Hbdiff], ΔTSI, and Δ[tHb] decreasing toward end of the test. Absolute maximal TSI values are shown in **Figure 4**, with a main effect of hypoxic condition and a continual decrease as hypoxia severity increased (*p* < 0.001) as well as a main effect of set duration near exhaustion (*p* < 0.05). No interactions were present.

### Cerebral oxygenation

**Figure 3:**
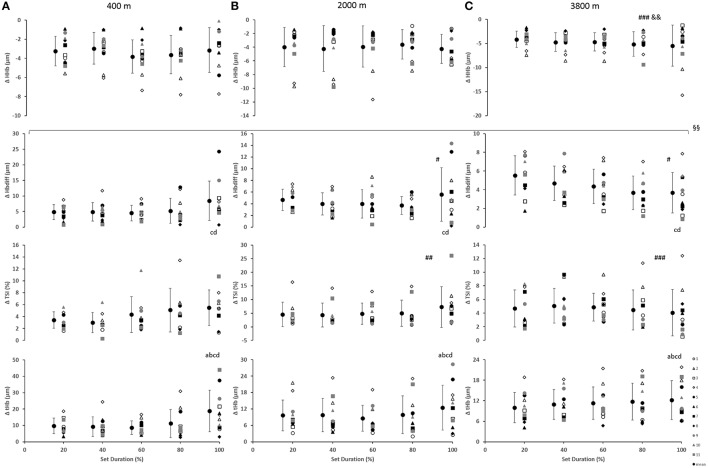
Near-infrared spectroscopy (NIRS) results representing the average maximum-minimum delta (Δ) value during the percentage of sprints completed to exhaustion for the individual response of the prefrontal cortex in simulated altitude of **(A)** 400 m, **(B)** 2000 m, and **(C)** 3800 m. Mean ± SD. ^###^*p* < 0.001, ^#^*p* < 0.05 for difference with 400 m; ^&&^*p* < 0.01 for difference with 2000 m. Symbol: a for difference with 20%, b for difference with 40%, c for difference with 60%, and d for difference with 80%. ^§§^Significant (*p* < 0.01) interaction (condition × set duration) was present.

In the prefrontal cortex (Figure 3), there was a main effect of condition for Δ[HHb] with a smaller decrease (smaller change) at 3800 m when compared with 400 and 2000 m (*p* < 0.01). Additionally, a main effect of condition resulted with greater Δ[Hbdiff] at both 2000 and 3800 m, compared with 400 m (*p* < 0.05). Similarly, there was higher ΔTSI at both 2000 and 3800 m when compared with 400 m (*p* < 0.01). There was a main effect of set duration near the end of the sprint test to exhaustion (*p* < 0.05) with Δ[Hbdiff] and Δ[tHb] increasing in the last 20% of the sprint test. In addition, for Δ[Hbdiff], there was a significant interaction effect between condition and set duration (*F* = 2.706, *p* < 0.01).

## Discussion

The main findings of this study were:

1. There was a continual decrease in convective factors of oxygen delivery (e.g., decreases in pulse oxygen saturation and peak oxygen uptake) with increased hypoxia severity, which was linked with impairment in performance (number of sprints and total work) across conditions.
2. There were reduced changes in peripheral oxygenation values at 3800 m indicating a possible limitation of the oxygen transport system (i.e., circulatory) which was non-linear as hypoxia increased, likely indicating different responses as altitude increases. This may suggest a threshold between 2000 and 3800 m regarding the means of oxygen transport, which supports previous research stating 2000 m moderate and 3800 m is high altitude (Bartsch et al., 2008).
3. Cerebral deoxygenation demonstrated greater changes at 3800 m compared with 400 and 2000 m, as well as an increased change in blood volume in the final 20% of the set duration (at exhaustion). This may indicate that central autoregulation occurs in order to continue exercise despite limited peripheral and cerebral oxygen delivery, until a certain point of limited diffusion at which protective mechanisms cease exercise.

### Decreased convective oxygen delivery

With increased altitude, there was an expected and logical decrease in convective factors of oxygen transport. Specifically, there was a decreased pulse oxygen saturation (level of the capillary) and decreased peak oxygen uptake (pulmonary and systemic circulation) with conditions of decreased oxygen. Maximal heart rate was also decreased significantly at 3800 m. Interestingly, there was no increase with altitude in minute ventilation or breathing frequency. However, since the exercise was maximal and to exhaustion, any difference between altitude levels would have been minor. The ventilatory efficiency index ($\overset{\cdot }{\text{V}}$_E_/$\overset{\cdot }{\text{V}}$O_2_) was increased with altitude naturally due to a decrease in oxygen uptake (Table 1), similar as previously shown (Calbet et al., 2009). It has been suggested that the first limitation of performance in hypoxia is the delivery of oxygen primarily by factors of convection (which resulted in the present study) and subsequently by means of diffusion capacities at the muscle and mitochondrial (Calbet et al., 2009, 2015b) as well as pulmonary level (Sarkar et al., 2017). Additionally, there were no differences in the hemodynamic response with repeated sprints to exhaustion between altitude conditions (SV, Q, HR, SVR, EDV, EF) and also no acute peripheral vasodilation as noted by femoral arterial blood flow. Peak cardiac output has been shown to remain similar between different levels of hypoxia as a possible counteraction for the reduction in arterial content of oxygen (Calbet et al., 2009). Furthermore, this study demonstrated that the limitation of oxygen delivery resulted in an impairment in performance across conditions. There were no changes in the rating of perceived exertion of the leg or breathing or changes in blood lactate concentration between conditions, indicating that the maximal efforts of the tests were similar despite the differences in total work. These results suggest there are some factor(s) allowing performance to be at least partly maintained despite continued decreases in pulse oxygen saturation and oxygen uptake due to regulatory mechanisms facilitating the oxygen delivery as the severity of hypoxia increases. This can be partly explained by the oxygen-hemoglobin dissociation curve and the shift of the curve based on the affinity for oxygen. As demonstrated by Calbet and colleagues, the offloading of oxygen from hemoglobin does not require a right-shift in the oxygen-hemoglobin dissociation curve indicating less of a role of the Bohr effect (Calbet et al., 2015b).

### Limitations of peripheral oxygenation

**Figure 4:**
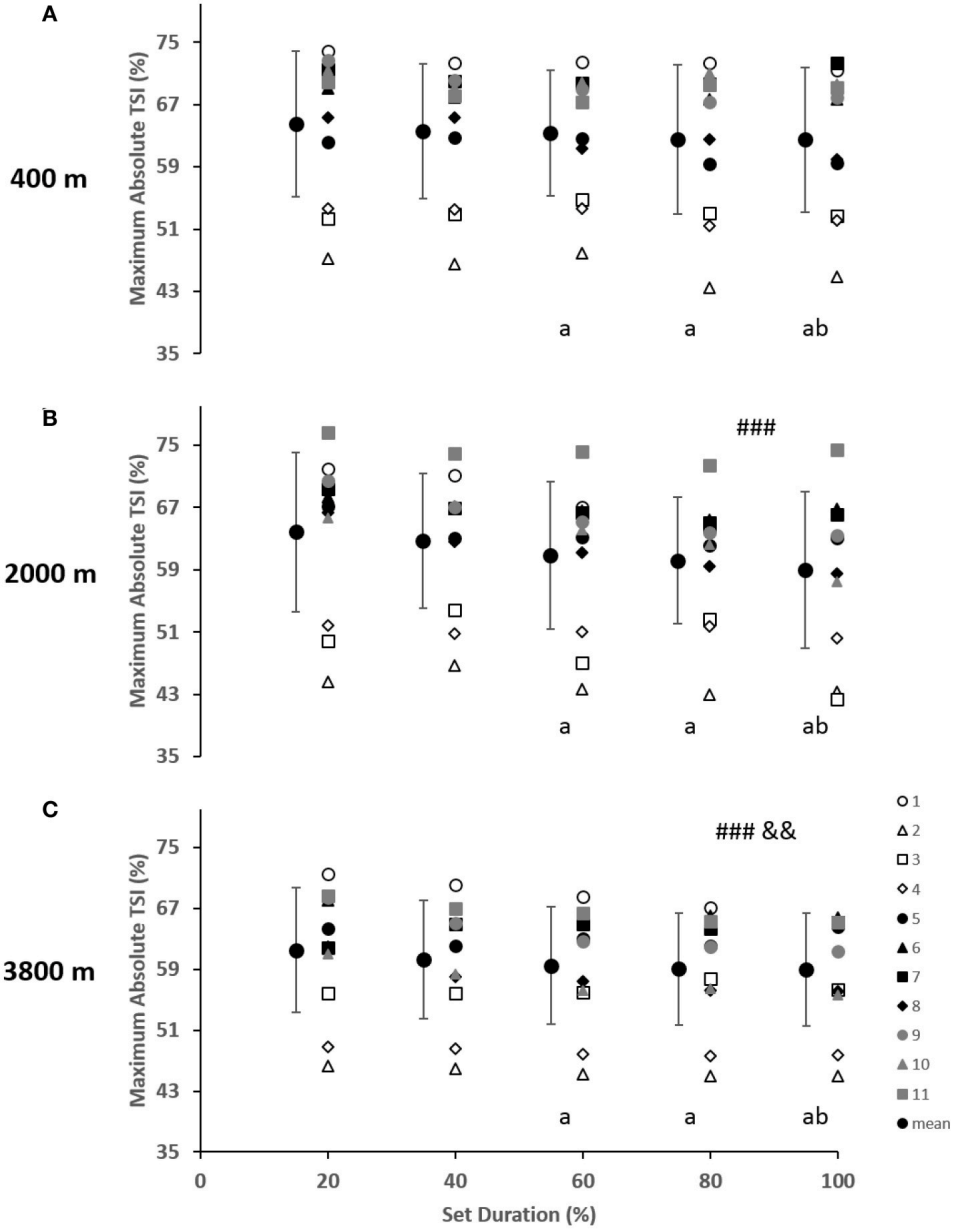
Near-infrared spectroscopy (NIRS) results representing the average maximum value of tissue saturation index (%) during the percentage of sprints completed to exhaustion for the individual response of the vastus lateralis in simulated altitude of **(A)** 400 m, **(B)** 2000 m, and **(C)** 3800 m. Mean ± SD. ^*###*^*p* < 0.001 for difference with 400 m; ^&&^*p* < 0.01 for difference with 2000 m. Symbol: a for difference with 20%, and b for difference with 40%.

The change in vastus lateralis oxygenation values were less at 3800 m indicating less of a change from maximum to minimum during sprints, meaning there was a further limitation of the oxygen transport system (i.e., circulatory) in comparison to the other conditions of 400 and 2000 m (Figure 2). Indeed, the absolute maximal values of TSI indicated a continual limitation of oxygen as hypoxia increased, which decreased toward the end of the test (Figure 4). In fact, an inconsistent decrease in Δ[HHb] was demonstrated as the severity of hypoxia increased, indicating a possible threshold between 2000 and 3800 m where peripheral oxygenation becomes limited. An increase in [HHb] in hypoxia has been shown to increase the metabolic demand of exercise (Costes et al., 1996) and is considered as a counteraction for the reduced oxygen availability (Legrand et al., 2005). This is likely due to increased local acidosis reducing hemoglobin's affinity for oxygen through the Bohr effect (Nielsen et al., 2002). Furthermore, previous research has demonstrated that the role of this acidosis is minimal regarding the mechanisms of oxygen offloading during exercise, which supports the result of the current study with no change in blood lactate concentration between conditions, as seen in Table 1 (Calbet et al., 2015b).

All changes in peripheral oxygenation (Δ[HHb], Δ[tHb], and ΔTSI) were continually decreasing toward the end of the test (Figure 1). In the present study, there was a plateau in Δ[HHb], which has been previously suggested to be an indication of maximal skeletal muscle oxygen extraction (Esaki et al., 2005). However, it has also been shown that the peripheral muscle can continue to extract oxygen even in conditions of reduced oxygen availability (Smith and Billaut, 2010, 2012). This supports previous research that suggested the plateau in peripheral deoxygenation was not a reason for the exhaustion and the end of exercise, but rather an indication of an equilibrium between the oxygen delivery and extraction/consumption over higher work rates (Subudhi et al., 2007). This confirms the result of the current study that performance can continue despite limited convective oxygen transport (decreased oxygen uptake and pulse oxygen saturation) as well as despite maximal levels of peripheral deoxygenation. The decreased Δ[HHb] in the vastus lateralis at 3800 m in the present study suggests that a compensatory larger extraction may at least partly counterbalance the lower convective oxygen supply and therefore reduce the diffusion limitation, as previously suggested (Cerretelli, 1976; Wagner, 2000).

Moreover, Amann and colleagues have suggested that when substantial peripheral muscle fatigue has occurred in normoxia or moderate hypoxia, there is no capacity to reverse the magnitude and thus, exercise is terminated via reduction in the central motor output for prevention of further development of peripheral fatigue beyond a critical level (in accordance with their previous research; Amann et al., 2006, 2007; Romer et al., 2007). In addition, researchers have suggested an influence of the central nervous system over the active muscles in hypoxia (Amann et al., 2007), as there is decreased central command at low oxygen fraction (Millet et al., 2009). This mechanism could partially contribute to the greater performance detriments with lower oxygenation levels (range of 70–75% arterial oxygen saturation), which is likely regulated by differences in the partial pressure of arterial oxygen (P_a,O2_) rather than changes in arterial content or hemoglobin concentration (Horstman et al., 1980; Calbet et al., 2002; Lundby and Damsgaard, 2006).

### Limitations of cerebral oxygenation

Greater decreases in prefrontal cortex deoxygenation (Δ[HHb]) were demonstrated at 3800 m. Cerebral changes in oxygenation were limited (lower) at both 2000 and 3800 m, in comparison to the 400 m condition. Furthermore, greater changes in [tHb], a well-known indicator of regional blood volume and perfusion (Van Beekvelt et al., 2001; Faiss et al., 2013), and Δ[Hbdiff] were seen at the end of the test (i.e., exhaustion, last 20% of sprints) with increased changes at both 2000 and 3800 m for Δ[Hbdiff]. These data together illustrate the oxygen delivery regulation of the brain.

Previous research has indicated that in hypoxic conditions there is a greater diffusive limitation due to a lower gradient of the partial pressure of oxygen (Wagner, 1993). In hypoxia, the brain adjusts for a lower arterial oxygen content by increasing the extraction of oxygen (Gonzalez-Alonso et al., 2004; Rasmussen et al., 2007) and increasing cerebral blood flow at rest (Lassen, 1959; Willie et al., 2012). While during exercise, there are the additional factors of hypocapnia and increased arterial blood pressure which contribute to the impact of oxygenation and brain function in hypoxia (Curtelin et al., 2017). Curtelin et al. (2017) found that the priority is placed on maintaining cerebral oxygen delivery even though there is also a need to regulate cerebral blood flow to avoid an excessive increase in blood pressure. During sprint exercise in hypoxia, the combination of increased mean arterial pressure and cardiac output increases perfusion of the muscle tissue while simultaneously challenging the brain with the risk of hyperperfusion (Bill and Linder, 1976; Deegan et al., 2010). As demonstrated in the present study, there were greater changes in the perfusion (Δ[tHb]) in the end of the test, which was likely tolerated despite higher perfusion pressure and reduced partial pressure of arterial carbon dioxide (P_a, CO2_) during sprint exercise in severe hypoxia in order to protect brain function in low oxygen conditions with the counter-risk of increased hemodynamic injury (Curtelin et al., 2017). This change was mostly present in the normoxic condition, however, there was a main effect difference at the end of exercise. Further, this finding may provide insight to the reason for exercise cessation. Moreover, the evidence of previous research showing a 66% decrease in cerebral P_a,O2_ with acute hypoxia cannot be ignored (Calbet et al., 2009). In fact, it was found that the low P_a,O2_ may be as low as 10 Torr in some areas of the brain (Calbet et al., 2003). This low P_a,O2_ would suggest there is a low pressure gradient, however it remains unknown about the diffusion distance and the possible diffusive limitation of oxygen delivery to the brain at altitude (Hornbein et al., 1989; Dunn et al., 2016). There is likely a coupling between arterial blood pressure and perfusion pressure (gradient between arterial and venous blood pressure) related to pressure, flow, and resistance to determine the mechanisms of the diffusion limitation of cerebral oxygen delivery during exhaustive exercise in hypoxia.

The results of the present study confirm that exercise performance in hypoxia can continue despite limited peripheral convective oxygen delivery, with the continuation of performance due to a functional reserve in muscle oxygen diffusing capacity (Calbet et al., 2015b). Meanwhile, the brain increases blood flow to account for decreased arterial oxygen content and to maintain oxygen delivery to preserve the brain's function (central motor output) and thus task continuation despite the progression of central fatigue (Amann et al., 2007; Curtelin et al., 2017). Progressive changes continue until an underlying threshold is reached where the pressure of increased perfusion of the brain surpasses a critical threshold and likely leads to exercise cessation due to increased mean arterial pressure and hyperperfusion. However, these mechanisms are speculative and therefore further research is warranted.

### Limitations of the study

Researchers acknowledge that the current results are influenced by an elevation of skin blood flow during exercise, which is due to the nature of the device placed on the surface of the skin (Subudhi et al., 2007). Therefore, it is probable that the current results would underestimate tissue deoxygenation. Moreover, it is likely that the changes found would be greater in these variables and would not alter the conclusions made here. The difference between hemoglobin and myoglobin is not known with the use of the NIRS, it is therefore not possible to distinguish the differences between blood-muscle oxygen transport in the current study. Furthermore, the reliability of probe placement was minimized with permanent markings and picture identification, however, there is day-to-day variation in these parameters of about 8.0–9.4% (Kishi et al., 2003; Kolb et al., 2004). It is assumed that [O_2_Hb] and [HHb] are in existing equilibrium where an increase in one simultaneously decreases the other. This assumption is based on maintaining [tHb] constant. As most of the body's arterial blood volume is oxygenated, [tHb] is closely related to [O_2_Hb] if the consumption of oxygen remains constant, which can be reflected by HHb concentration (Van Beekvelt et al., 2001; Subudhi et al., 2007). During exercise, these variables are in constant flux and for that reason, the NIRS measurements should be considered with scrutiny. In addition, the test-retest reliability of the blood flow measurements from the femoral artery conducted pre- and post-RSAT have not been previously reported, thus it is important to consider in interpretation of these results. As this study involved non-invasive measurements as part of a larger protocol, there is a lack of data corresponding to changes in blood gases, blood pressure, and cerebral blood flow which would certainly improve the interpretation of these results.

## Conclusion

In summary, this study confirms that performance in hypoxia is limited by continually decreasing convective oxygen delivery while exercise can continue despite maximal peripheral deoxygenation. The limitation of peripheral oxygenation was not linear across varying hypoxic conditions possibly indicating a threshold at which point oxygen delivery has a stronger effect on the periphery between 2000 and 3800 m. There may be a cerebral autoregulation that increases cerebral perfusion at exhaustion to account for decreased arterial oxygen content (as shown across all conditions) and allow for task continuation (greater cerebral deoxygenation at 3800 m in comparison to 400 and 2000 m).

## Author contributions

All authors listed have made substantial, direct, and intellectual contributions to this work. In addition, all authors have approved this work for publication.

### Conflict of interest statement

The authors declare that the research was conducted in the absence of any commercial or financial relationships that could be construed as a potential conflict of interest.

## Abbreviations

F_I_O_2_: fraction of inspired oxygen
NIRS: near-infrared spectroscopy
Δ: delta change over time
RSAT: repeated sprint ability test
$\overset{\cdot }{\text{V}}$O_2_: maximal oxygen uptake
$\overset{\cdot }{\text{V}}$_E_: minute ventilation
$\overset{\cdot }{\text{V}}$_E_/ $\overset{\cdot }{\text{V}}$O_2_: ventilatory equivalent for oxygen
$\overset{\cdot }{\text{V}}$_E_/ $\overset{\cdot }{\text{V}}$CO_2_: ventilatory equivalent for carbon dioxide
RER: respiratory exchange ratio
BF: breathing frequency
SV: stroke volume
HR: heart rate
Q: cardiac output
SVR: systemic vascular resistance
EDV: end diastolic volume
EF: ejection fraction
RPE: rate of perceived exertion
O_2_Hb: oxyhemoglobin
HHb: deoxyhemoglobin
tHb: total hemoglobin
Hbdiff: hemoglobin difference
TSI: tissue saturation index
P_a,CO2_: partial pressure of arterial carbon dioxide
P_a,O2_: partial pressure of arterial oxygen.
